# CHANGE IN OLDER ADULTS’ SELF-EFFICACY TO HANDLE DISASTERS AFTER A PREPAREDNESS PROGRAM: ROLE OF SOCIAL NETWORKS

**DOI:** 10.1093/geroni/igac059.2654

**Published:** 2022-12-20

**Authors:** Saida Jaboob, Lena Thompson, Abinaya Ilavarasan, Sato Ashida

**Affiliations:** University of Iowa, Coralville, Iowa, United States; University of Iowa, Iowa City, Iowa, United States; University of Iowa, Iowa City, Iowa, United States; University of Iowa College of Public Health, Iowa City, Iowa, United States

## Abstract

Social support networks provide a context within which older adults maintain their safety and well-being. Preparing for disasters and emergency situations can help older adults stay safe independent after a disaster. Self-efficacy to prepare for and handle disaster situations can influence how older adults proactively prepare and protect themselves. Fifty-four older adults 61 to 92 years of age in Eastern Iowa were interviewed before and after participating in an intervention program that helped them develop personalized disaster management plans. Participants reported seven support network members on average, ranging from one to 23. About one-third of the network members were participants’ children and grandchildren, 11% were their siblings and parents, and 58% were non-family members such as friends and neighbors. Out of 549 network members identified, 245 were selected as someone participants can depend on during emergency situations; 47% were family whereas 43% were non-family members. Participants who reported higher numbers of network members whom they can depend on in emergency situations at baseline showed more increase in self-efficacy to manage disasters one-month after receiving the intervention (p=0.02). Having social support network members whom older adults feel they can turn to during emergency situations may help boost their confidence in handling and preparing for disasters through participating in a disaster preparedness program. Participants indeed identified additional members whom they could depend on in disaster situations after the intervention. Future studies may test strategies to enhance social support networks to increase confidence among older adults to prepare for and handle disasters.

